# Intravitreal dexamethasone implant for central retinal vein occlusion without macular edema

**DOI:** 10.1186/s12886-019-1097-y

**Published:** 2019-04-17

**Authors:** Eun Young Choi, Hyun Goo Kang, Sung Chul Lee, Min Kim

**Affiliations:** 1 Department of Ophthalmology, Gangnam Severance Hospital, Yonsei University College of Medicine, 211, Eonjuro, Gangnam-gu, Seoul, 06273 South Korea; 2 Department of Ophthalmology, Severance Hospital, Yonsei University College of Medicine, 50-1, Yonseiro, Seodaemun-gu, Seoul, 03722 South Korea

**Keywords:** Central retinal vein occlusion, Intravitreal dexamethasone implant, Ozurdex, Steroid, Fundus pathomorphology

## Abstract

**Background:**

To evaluate the efficacy of an intravitreal dexamethasone (IVD) implant (Ozurdex®) for the treatment of central retinal vein occlusion (CRVO) without macular edema (ME).

**Methods:**

A retrospective cohort study was designed, and 20 eyes of 20 patients diagnosed with non-ischemic CRVO without ME were included. A total of 10 CRVO eyes were observed without treatment, and another 10 CRVO eyes received a single IVD injection at baseline. Mean changes in pathomorphologic parameters of fundus and optical coherence tomography parameters were measured at baseline and at 1, 3, 6, and 12 months.

**Results:**

The decreases in venous tortuosity (*p* = 0.014 for superior; 0.036 for inferior arcades) and width (*p* = 0.024 for superior; 0.003 for inferior arcades) from baseline to 12 months after injection were significantly greater in the treated group than the observed group. The improvements in RNFL swelling (*p* = 0.010) and retinal hemorrhage (*p* = 0.006) were also significantly greater in the treated group. Visual symptom improvement was significantly faster in the treated group (*p* = 0.001). In two cases, IVD injection resulted in complete resolution of cilioretinal artery occlusion associated with the CRVO, leading to complete visual recovery in 1 week. None of the treated eyes showed signs of ME development, ischemia progression, or neovascularization.

**Conclusions:**

IVD implant was significantly effective in improving venous engorgement, retinal hemorrhage, RNFL swelling, and visual symptoms by presumed alleviation of disc swelling and venous outflow. This treatment may be a considerable treatment option in CRVO patients with no ME.

## Background

Central retinal vein occlusion (CRVO) is caused by a venous outflow blockage in the main trunk of the central retinal vein. It can result in severe vision loss due to macular edema (ME), intraretinal hemorrhage, and ischemia leading to neovascularization [1–3]. Unlike branch retinal vein occlusion, the precise CRVO blockage site cannot be easily seen in the retina, as it is thought to occur within the optic nerve. The etiology of CRVO is not clearly known, and is thought to result from a combination of multiple factors. CRVO in elderly individuals may be due to abnormally increased arterial stiffness affecting neighboring veins [4, 5]. Some have concluded that the compact anatomy of optic nerve head (ONH) may play a role in the pathogenesis of CRVO [6–8]. Additionally, inflammation of the central retinal or peripapillary vein has been proposed as a possible cause, especially in young adults [9, 10].

The prognosis of CRVO can differ depending on its angiographic subtype: either ischemic or non-ischemic, and the primary cause of poor visual outcome in ischemic CRVO is ME [11]. Natural clinical courses of the two CRVO types are completely different: outcome is much better in non-ischemic CRVO than in ischemic CRVO. However, more than 2/3 of non-ischemic CRVO patients showed vision and visual field deterioration at their 3-month follow-up assessment [12]. Resolution of retinal venous engorgement was seen within only 1 year in 17.3% of non-ischemic CRVO with or without ME [13]. Optic disc edema also remained in approximately 20% of cases, even after 24 months [13]. Moreover, the Central Vein Occlusion Study (CVOS) [14] reported that although over 75% of CRVOs are non-ischemic, 34% of those cases converted to the ischemic form during 3-year follow-up. In the CVOS study [2], up to 60% of eyes with ischemic CRVO developed neovascularization, usually in the anterior segment, with neovascular glaucoma developing in one-third of all ischemic CRVO cases.

In the treatment of CRVO with ME, the GENEVA study [15] revealed that intravitreal steroid therapy could significantly improve ME and visual outcomes. However, for CRVO patients without ME complaining of severely decreased visual quality or newly developed blind spots, there is no currently recommended treatment available for now. Frequent follow-up is the only recommendation, which monitors the development of ME, neovascularization, and conversion to ischemic CRVO, which can cause serious impairments to the patient’s visual outcomes. Edematous change of ONH associated with unknown inflammatory reaction leading to compression of CRV have been suggested as possible causes of CRVO [9, 16]. Therefore, use of intravitreal steroids as a possible treatment could be considered due to the inflammatory pathogenic mechanism of CRVO, even in eyes without ME [17, 18]; however, there has been no clinical study investigating the efficacy of intravitreal steroid treatment in CRVO eyes with no ME. We hypothesize that intravitreal dexamethasone (IVD) implant (Ozurdex®; Allergan Inc., Irvine, CA, USA) treatment will act to relieve ONH swelling by providing an anti-inflammatory effect, thereby alleviating central venous outflow. Therefore, we retrospectively compared clinical outcomes between IVD-injected and untreated patients to investigate the potential therapeutic effect of IVD implant in CRVO without ME.

## Methods

### Study design and participants

We designed a retrospective cohort study to evaluate the anatomical and functional effectiveness of IVD implant treatment in CRVO eyes. This study was carried out at a single center – Gangnam Severance Hospital, Seoul, Korea - by two retina specialists (E.Y.C, M.K.). Study subjects were collected through a review of medical records from February 2012 to April 2017. This study was approved by the Institutional Review Board at Gangnam Severance Hospital (IRB approval number: 3–2017-0112). We did not obtain patient consent, since data were analyzed anonymously. Only patients with definitive diagnosis of non-ischemic CRVO without ME were included in this study. CRVO patients with signs of inner retinal ischemia (e.g., ocular neovascularization, 10 disc areas or more of retinal capillary non-perfusion, or relative afferent pupillary defect) were excluded. We excluded patients who had been treated by intravitreal drug injections or laser photocoagulation before the baseline study. Patients with other retinal disorders (e.g., diabetic retinopathy or age-related macular degeneration), optic nerve diseases (e.g., optic neuritis, glaucoma, or ischemic optic neuropathy), or uveitis were also excluded.

We reviewed the medical records of 29 eligible patients who had non-ischemic CRVO but no ME. Nine cases were excluded from the analysis due to incomplete data. One-year follow-up was not completed in four cases. In the other five cases, some of the tests required for analysis were not performed. Ozurdex® was approved by the Korea Ministry of Food and Drug Safety in November 2011 for the treatment of ME following retinal vein occlusion, and it was actually applied in 2013 by our institution. IVD implant therapy was administered to all CRVO without ME patients who agreed to treatment since January 2013, except for two patients who refused treatment. Before that time, CRVO eyes with no ME were usually observed without any treatment. There were no additionally required conditions regarding the decision to treat.

Patients were divided into the following two groups according to their treatment history for comparative analysis: untreated observation group and IVD implant-treated group. Treatment history of each patient was unknown to the study investigators who collected patient data and assessed outcomes.

The observation group patients were followed up without any treatment, and the IVD-treated group patients received a single injection within 1 week after the onset of symptoms. Every IVD implantation was performed by a single retina specialist (M.K.) following the routine injection protocol: topical anesthesia (0.5% proparacaine) was applied and 5% betadine solution was placed into inferior fornix after lid scrub with povidone-iodine. Sustained-release dexamethasone (Ozurdex®) (0.70 mg) was administrated 3.0–3.5 mm posterior to limbus through the pars plana using a sterile technique.

### Study outcomes

The primary outcome was the mean changes in pathomorphologic parameters of the fundus (such as vessel tortuosity, central retinal vein width, retinal hemorrhage amount, and cotton wool spots), and spectral-domain optical coherence tomography (SD-OCT) parameters (such as central macular thickness, subfoveal choroidal thickness, and retinal nerve fiber layer [RNFL] thickness of the optic nerve head). For secondary outcomes, we compared the final visual acuity and the time point when visual symptoms had improved.

**Fig. 1:**
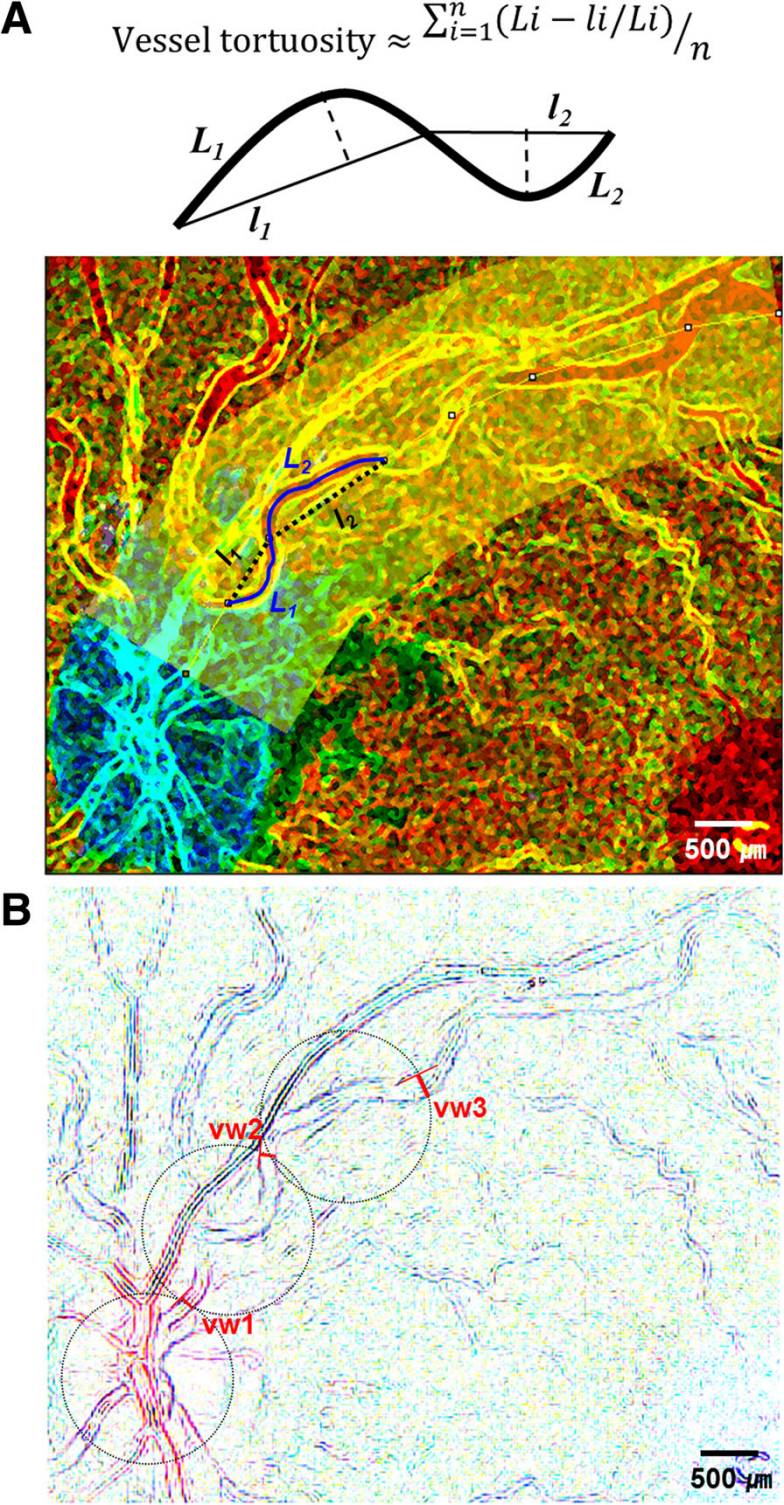
Illustration of the subdivision used to estimate vessel tortuosity (**a**) and vascular width (**b**). Magnified (3×) fundus photo images were used for the analysis, and images showing the subdivided superior central retinal vein are presented. With imageJ software (Version 1.47, NIH, Maryland, USA), image was processed twice: sharpening to enhance the vessel cords for tortuosity analysis (**a**) and reversal to highlight the vessel walls for width analysis (**b**). Vessel tortuosity was estimated with relative length variation (refer to equation of top) by using straight and freehand line selection tools (**a**). Vascular width was measured at three points: at disc margin at 1.0 disc diameter from the margin, and at 2.0 disc diameter from the margin by using straight line selection tool (**b**). *L* = curvilinear length of the vessel; *l =* linear length of the chord; vw = vascular width

The results of ophthalmic evaluations were collected at the initial visit and at follow-up times of 1 month, 3 months, 6 months, and 12 months after either the initial visit or IVD treatment. The collected data included best-corrected visual acuity (BCVA, logMAR) and intraocular pressure (IOP, mmHg), results of detailed anterior segment and fundus examinations, fundus photography, wide field fundus imaging, SD-OCT, and fluorescein fundus angiography. Whether the patient’s visual symptom had improved or not was also evaluated. Fundus photography, fluorescein angiography, and wide-field fundus imaging each captured images of 30°, 55°, and 200° of the retina. SD-OCT images were obtained with Heidelberg SD-OCT (Spectralis®; Heidelberg Engineering, Heidelberg, Germany), and included enhanced depth imaging to evaluate the deep choroid layers. Both fundus photography and wide field fundus images were used for the analysis of central retinal veins (CRV), presence of retinal hemorrhage, and cotton wool spots. Vessel tortuosity of superior and inferior CRV was measured as the relative length variation, defined as the integral of curvature normalized by the total path length using the formula shown in Fig. 1 [19–21]. Vascular analysis was limited to within the extent visible in the central retina (approximately 6 mm around the fovea). Vessel length was measured using ImageJ software (Version 1.47, NIH, Maryland, USA). Vessel widths of superior and inferior CRV were measured using the diameter plug-in of ImageJ software, which was the average of three points: at the disc margin, at 1.0 disc diameters (DD), and at 2.0 DDs from the margin (Fig. 1). Retinal hemorrhage was subjectively graded as 0 = no hemorrhages, 1 = minimal/small hemorrhages, 2 = medium amount of hemorrhages, or 3 = extensive hemorrhages in six divided fundus regions: the macular region, remaining posterior pole, and peripheral region divided into four sub-areas. [13] The sum of the weighted hemorrhage scores was calculated using the following: grade × 10 in the macular region, × 5 in the rest of posterior pole, and × 1 in a quarter of the peripheral region. Cotton wool spots were evaluated by the total number in the central retina.

Superior and inferior RNFL thickness of ONH were measured automatically by the built-in software (HEYEX PACS™ Version 1.8) of Heidelberg OCT viewer. Thickness of the central macula was measured automatically, and choroidal thickness was measured manually by using the caliper tool provided by the same software, measuring the distance from the retinal pigment epithelium to the chorioscleral junction at the subfoveal center. All tests and measurements were performed by two masked observers (E.Y.C, H.G.K.). The averaged values of the results obtained by two masked observers were used for analysis. No measurement was excluded due to a serious discrepancy between the two observers.

### Sample size and statistical analysis

Since the prevalence of CRVO was low (< 0.1 to 0.3%) [3, 22–24] and this study was conducted as an exploratory study to investigate the therapeutic effect of IVD implant only in CRVO eyes without ME, the number of subjects was determined as the minimum. Therefore, by using the decision-theoretic approach, the estimated sample size was 9, with a relatively high assumed mean response rate for IVD treatment, 0.85 and with low prior weight, 2.

A Bland-Altman assessment was used to assess the agreement of the measurements by two observers. A range of agreement was defined as mean bias ±2 standard deviation [25]. There was no measurement which showed a clinically important discrepancy. The averaged values of the results obtained by two masked observers were used for the analysis. Means ± standard deviations are presented for continuous variables, and Mann-Whitney U test was used to compare the groups at each time point. Fisher’s exact test was used for categorized parameters, which are expressed as frequencies. Changes in continuous variables between baseline and endpoints were analyzed using Wilcoxon signed rank test. The impact of IVD implant injection on the course of pathomorphological changes in the fundus was analyzed by the generalized estimating equation according to the data distribution pattern. SPSS software (version 23.0, SPSS, Inc.) was used for statistical analysis. A *p*-value less than 0.05 was considered statistically significant. All statistical tests were two-sided at the 95% confidence interval.

## Results

Ten observed CRVO eyes and another 10 IVD-treated CRVO eyes were analyzed in each group and compared to each other. All subjects were followed up for at least 1 year. There were no injection-related serious adverse events reported, including infection, glaucoma, and cataracts. Table 1 shows the baseline characteristics of the patients at the first visit or prior to the first IVD implant treatment. There were no significant differences in age, sex, BCVA, IOP, spherical equivalents, and the mean time since onset of disease between the two groups. Systemic diseases such as hypertension and type 2 diabetes mellitus were found more frequently in the observation group than in the IVD-treated group, but the difference was not statistically significant. No patient had a history of hypercoagulopathy or systemic autoimmune/inflammatory diseases.

**Table 1:** Baseline clinical characteristics of patients with central retinal vein occlusion without macular edema

	Observation group	IVD-treated group	*p-*value
Patients/ eyes (N)	10/10	10/10	N/A
Sex, female/ male^b^ (N)	9/2	7/2	0.38
Involved eye, right/ left^b^ (N)	6/5	3/6	0.67
Mean age^a^ (y)	51.7 ± 20.7	43.2 ± 12.2	0.15
Hypertension^b^ (N (%))	3 (27.3)	1 (11.1)	0.36
Type 2 diabetes^b^ (N (%))	2 (18.2)	1 (11.1)	0.88
Hypercoagulopathy (N (%))	0 (0)	0 (0)	N/A
Systemic autoimmune/inflammation (N (%))	0 (0)	0 (0)	N/A
Mean BCVA^a^ (logMAR)	0.11 ± 0.23	0.12 ± 0.33	0.61
Mean IOP^a^ (mmHg)	14.3 ± 2.0	12.0 ± 3.2	0.44
Spherical equivalent^a^ (diopter)	−0.60 ± 0.53	−0.89 ± 0.34	0.27
Mean duration of the disease^a^ (weeks)	3.6 ± 4.6	3.8 ± 3.3	0.06

Values are presented as mean ± standard deviation

*IVD* intravitreal dexamethasone, *BCVA* best-corrected visual acuity, *IOP* intraocular pressure

^a^Mann-Whitney U test and ^b^Fisher’s exact test were used for analysis

**Fig. 2:**
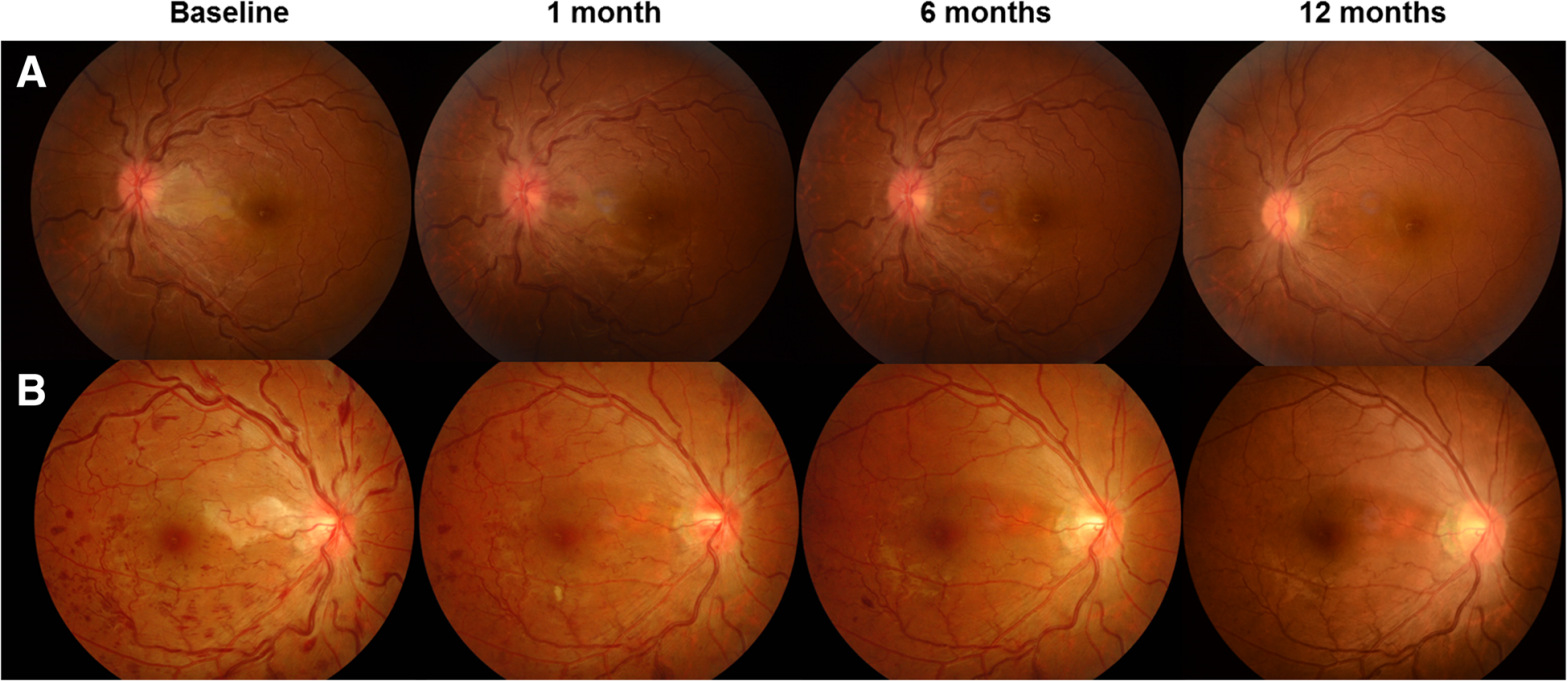
Representative figures at each time point of two cases of Ozurdex-treated central retinal vein occlusion (CRVO) eyes with cilioretinal artery occlusion. A patient between the ages of 25 and 29 years (**a**) and a patient between the ages of 35 and 39 years (**b**) with CRVO and cilioretinal artery occlusion in their left and right eye each. Disc swelling and cilioretinal artery occlusion were significantly improved at 1 month after injection (**a**). Severe tortuosity and congestion of central retinal veins decreased gradually, and were nearly resolved at 1 year follow-up (**a**). Dramatic Improvements in disc swelling, vascular changes, and severe retinal hemorrhages were noted only 1 week later the treatment, and the favorable condition remained well until 1 year (**b**)

In two patients, IVD injection resulted in complete resolution of cilioretinal artery occlusion associated with CRVO leading to complete visual recovery in 1 week. The first patient was a young woman in her late 20s (Fig. 2) who visited the emergency room because of sudden vision loss in her left eye. Her BCVA was 20/200, and no remarkable sign was noted in the anterior segment and vitreous. Fundus examination revealed a cilioretinal artery occlusion and moderate vascular tortuosity without any sign of ME. After 3 days, disc edema and vascular tortuosity became worse and a dense retinal hemorrhage appeared on the temporal side of the optic disc. There was no change in her VA. IVD treatment was performed on her left eye. A week later, her BCVA dramatically improved to 20/28. Disc swelling and cilioretinal artery occlusion were improved at 1 month after the injection, and her BCVA recovered to 20/20 at that time. The other case (Fig. 2) was a patient in her late 30s who visited our clinic due to an abrupt visual field defect in her right eye. Her BCVA was 20/25. Fundus examination revealed ischemic changes to the macular area supplied by the cilioretinal artery; additionally, multiple blot hemorrhages were found throughout the retina with tortuous retinal veins in her right eye. No other abnormal signs were observed. A week later, her BCVA was unchanged; however, a markedly increased number of retinal hemorrhages and cotton wool spots with severe disc swelling were noted, although there was no sign of ME. Her right eye was treated with IVD. Seven days later, her BCVA was recovered to 20/20, and improvements in disc swelling and retinal hemorrhages were noted. One month later, vascular tortuosity and retinal hemorrhages improved dramatically with almost complete resolution of cilioretinal artery occlusion and disc swelling. In both CRVO cases, an extensive laboratory workup associated with systemic coagulopathy and inflammatory/autoimmune conditions was performed, and erythrocyte sedimentation rate (ESR) elevation was confirmed.

### Longitudinal changes of pathomorphological parameters over a 1 year peroid

At baseline, fundus examinations revealed no significant differences between the groups (Table 2, *p* = 0.78). Superior vessel tortuosity significantly decreased from 0.34 ± 0.20 at baseline to 0.17 ± 0.07 after 1 year in the IVD-treated group (Table 2, *p* = 0.025), whereas patients in observation group experienced no significant change in superior vascular tortuosity measuring 0.24 ± 0.14 at baseline and 0.18 ± 0.05 after 1 year (Table 2, *p* = 0.094). Similarly, inferior vein tortuosity significantly decreased from 0.67 ± 0.45 at baseline to 0.18 ± 0.13 at 1 year (Table 2, *p* = 0.002) in the treated group, whereas in the untreated CRVO eyes, inferior vessel tortuosity showed no significant decrease within 1 year, from 0.50 ± 0.15 at baseline to 0.33 ± 0.15 (Table 2, *p* = 0.059). Interestingly, there was a statistical difference between the two groups regarding superior and inferior vascular tortuosity decrease (*p* = 0.014 and 0.036) from baseline to the last visit.

**Table 2:** Periodic changes in pathomorphologic findings from fundus

	Observation group		IVD-treated group		*p-*value
	Mean	SD	Mean	SD	
Vascular tortuosity (μm/μm)					
Superior					
Baseline	0.24	0.14	0.34	0.20	0.78*
1 month	0.24	0.09	0.25	0.06	0.90*
3 months	0.21	0.08	0.20	0.07	0.98*
6 months	0.19	0.07	0.18	0.07	0.87*
12 months	0.18	0.05	0.17	0.07	0.89*
*p-*value	0.094^†^		**0.025** ^**†**^		**0.014** ^**‡**^
Inferior					
Baseline	0.50	0.15	0.67	0.45	0.13*
1 month	0.46	0.18	0.40	0.31	0.26*
3 months	0.38	0.13	0.30	0.24	0.15*
6 months	0.34	0.12	0.30	0.30	0.59*
12 months	0.33	0.15	0.18	0.13	0.14*
*p-*value	0.059^†^		**0.002** ^**†**^		**0.036** ^**‡**^
Vascular width (μm)					
Superior					
Baseline	297.28	69.87	310.13	79.89	0.12*
1 month	288.45	68.61	266.94	71.33	0.020*
3 months	273.33	54.78	262.47	55.37	0.13*
6 months	270.71	47.81	252.53	51.64	0.39*
12 months	238.37	70.56	245.03	58.82	0.48*
*p-*value	**0.014** ^**†**^		**0.007** ^**†**^		**0.024** ^**‡**^
Inferior					
Baseline	285.30	68.63	295.05	88.79	0.13*
1 month	288.85	47.55	258.60	59.37	0.012*
3 months	250.22	49.02	236.21	50.38	0.062*
6 months	248.00	37.73	216.44	69.29	0.26*
12 months	227.01	49.21	213.20	48.21	0.22*
*p-*value	0.11^†^		**0.001** ^**†**^		**0.003** ^**‡**^
Retinal hemorrhage (score)					
Baseline	20.60	15.38	27.70	9.70	0.052*
1 month	19.62	11.63	12.50	10.30	0.20*
3 months	12.22	8.52	8.01	9.00	0.062*
6 months	11.20	8.24	7.40	5.86	0.18*
12 months	8.24	4.75	3.16	4.88	0.062*
*p-*value	**0.010** ^**†**^		**< 0.001** ^**†**^		**0.006** ^**‡**^
Cotton wool spots (number)					
Baseline	3.75	2.79	3.89	3.28	0.24*
1 month	2.75	2.10	2.11	3.10	0.67*
3 months	2.33	0.22	2.03	0.15	0.66*
6 months	2.12	0.38	2.01	0.34	0.74*
12 months	1.18	0.40	1.22	0.60	0.76*
*p-*value	**0.004** ^**†**^		**0.002** ^**†**^		0.42^‡^

Analysis used *Mann-Whitney U test to compare groups at each time-point; ^†^Wilcoxon signed-rank test to compare baseline and endpoints within a group; and ^‡^generalized estimating equation to compare changes with time between the groups

*IVD* intravitreal dexamethasone

The bold font for *P*-values indicates a statistical significance (i.e., *P* < .05)

Superior vascular width significantly decreased from 310.13 ± 79.89 μm at baseline to 245.03 ± 58.82 μm at 12 months in the IVD-treated group (Table 2, *p* = 0.007), and from 297.28 ± 69.87 at baseline to 238.37 ± 70.56 at 12 months in the observation group (Table 2, *p* = 0.014). Inferior vessel width also significantly decreased from 295.05 ± 88.79 μm at baseline to 213.20 ± 48.21 μm at 12 months in the treated group (Table 2, *p* = 0.001), but showed no significant decrease from 285.30 ± 68.63 at baseline to 227.01 ± 49.21 at 12 months in the observed group (Table 2, *p* = 0.11). A significant difference was found only at 1 month in both the superior and inferior central vein width (*p* = 0.020 and 0.012, respectively). The decrease from baseline to the last visit was significantly larger in the treated group than in the non-treated group for both superior and inferior vascular width (*p* = 0.024 and 0.003, respectively).

**Fig. 3:**
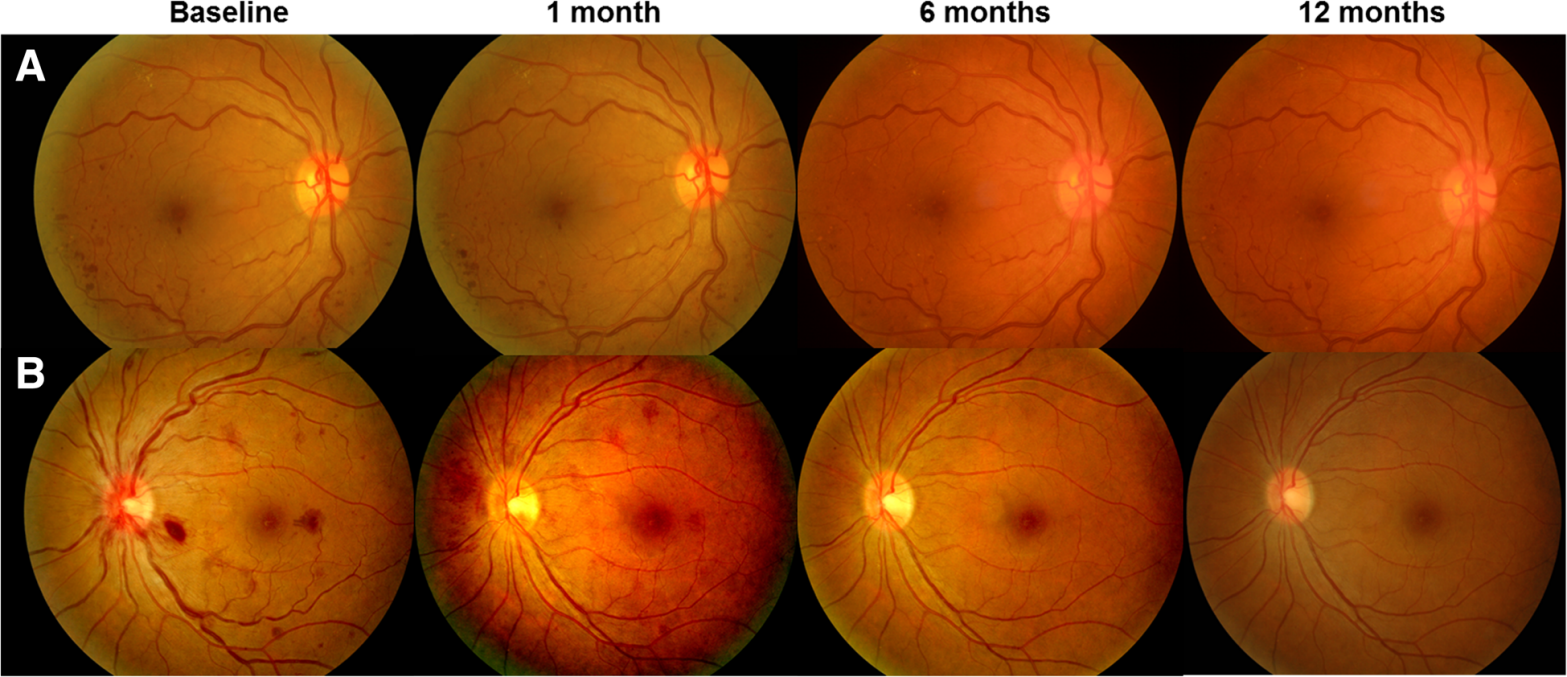
Representative figures of untreated (**a**) and Ozurdex-treated (**b**) central retinal vein occlusion eyes at each time point. Retinal hemorrhage was more extensive in (**b**) compared to (**a**) at baseline. In untreated eye (**a**), some dot hemorrhages were still found from 6 months to 12 months. However, no additional hemorrhagic signs were observed after 6 months in Ozurdex -treated eye (**b**). Vessel tortuosity was dramatically improved after 1 month in treated eye (**b**), while engorged vessels remained almost unchanged during the 12-month follow-up in untreated eye (**a**). Edematous change in the disc was more prominent in treated eye (**b**), and it gradually resolved throughout the follow-up period

The grades of retinal hemorrhage significantly decreased from 27.70 ± 9.70 at baseline to 3.16 ± 4.88 at 12 months in the IVD-treated group (Table 2, *p* < 0.001), and from 20.60 ± 15.38 at baseline to 8.24 ± 4.75 at 12 months in the observation group (Table 2, *p* = 0.010). However, the improvement in retinal hemorrhage grades was significantly larger in the treated group than in the non-treated group (Table 2, *p* = 0.006). The average numbers of cotton wool spots significantly decreased from 3.89 ± 3.28 at baseline to 1.22 ± 0.60 at 12 months in the IVD-treated group (Table 2, *p* = 0.002), and from 3.75 ± 2.79 at baseline to 1.18 ± 0.40 in the observed group (Table 2, *p* = 0.004). However, changes over time did not show significant differences between the groups (Table 2, *p* = 0.42). Figure 3 shows representative images of both groups at each time point. IVD-treated eyes had more prominent improvements in retinal hemorrhage, vascular tortuosity, and disc edema than in untreated eyes.

### Longitudinal changes of OCT parameters over 1 year

**Fig. 4:**
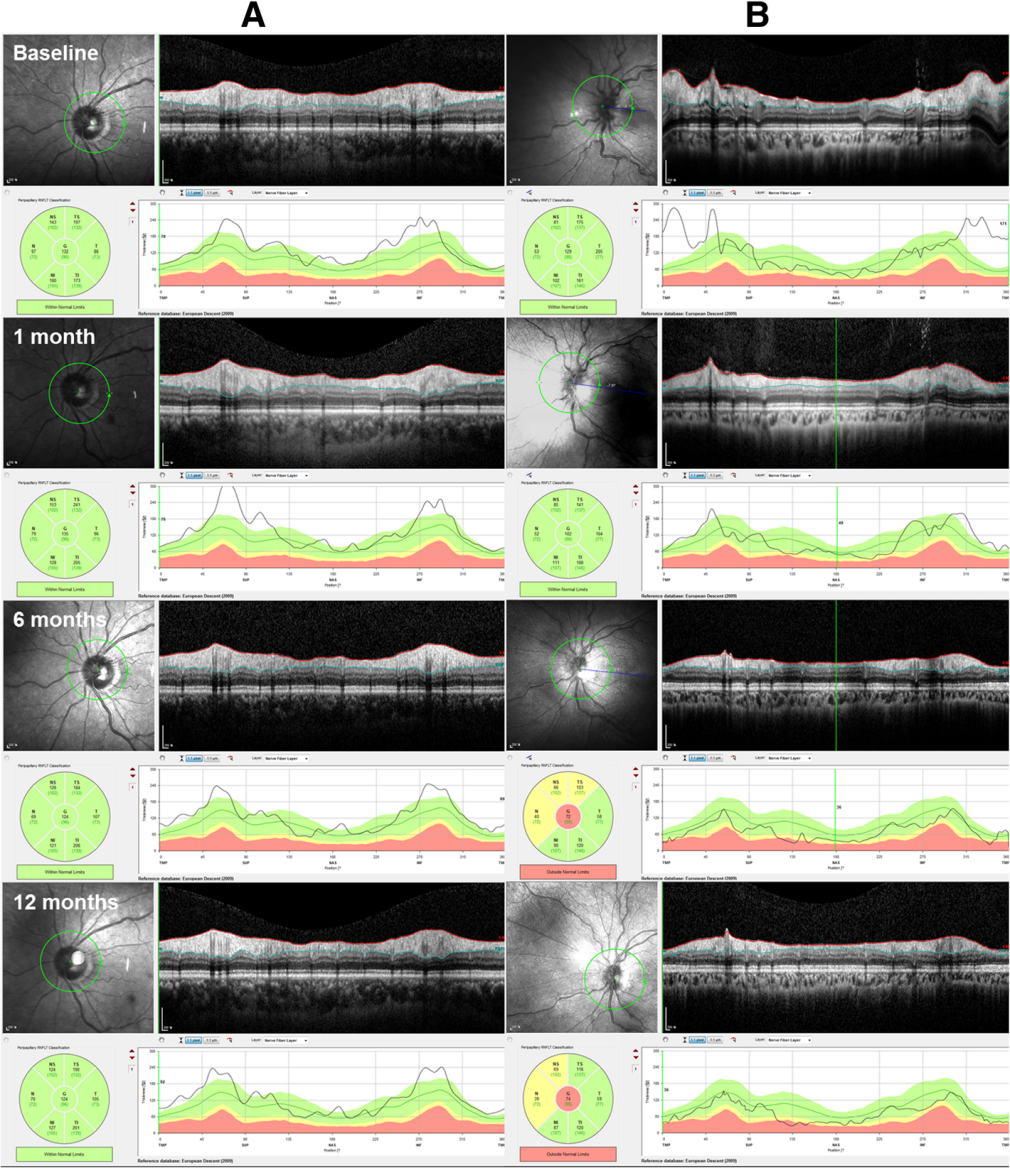
Representative optical coherence tomography figures analyzing peripapillary retinal nerve fiber layer (RNFL) thickness of the optic nerve head (ONH) untreated (**a**) and Ozurdex-treated (**b**) central retinal vein occlusion eyes at each time point. RNFL thickening of ONH was prominent in both untreated (**a**) and Ozurdex-treated (**b**) eyes at baseline. It became thicker at 1 month and slightly decreased thereafter, but thickness remained above the normal range for up to 12 months in untreated eye (**a**). Conversely, in Ozurdex-treated eye (**b**), a significant improvement was observed at 1 month, and thickness gradually returned to its normal range during the follow-up period

Peripapillary RNFL thickness decreased significantly from 238.66 ± 39.09 μm at baseline to 144.40 ± 10.90 μm in the IVD-treated group (Table 3, *p* = 0.023), though it showed no significant change over 1 year in the observation group (Table 3, *p* = 0.56). It was remarkable that IVD implant treatment caused significant improvement in disc swelling in CRVO eyes (Table 3, *p* = 0.010). When comparing RNFL thickness between the groups at each time point, statistically significant differences were noted at the 1- and 12-month evaluations, with the IVD-treated group achieving significantly greater improvement (*p* < 0.001 and *p* = 0.009, respectively; Table 3 and Fig. 4). Central macular thickness and subfoveal choroidal thickness revealed no significant changes during the follow-up period in both groups (Table 3).

**Table 3:** Longitudinal changes in parameters from spectral-domain optical coherence tomography

	Observation group		IVD-treated group		*p-*value
	Mean	SD	Mean	SD	
Peripapillary RNFL thickness (μm)					
Baseline	230.00	37.80	238.66	39.09	0.23*
1 month	287.00	36.59	186.00	59.90	< 0.001*
3 months	207.72	27.87	184.70	29.41	0.066*
6 months	195.25	58.65	177.42	41.20	0.062*
12 months	212.33	47.92	144.40	10.90	0.009*
*p-*value	0.56^†^		**0.023** ^**†**^		**0.010** ^**‡**^
Central macular thickness (μm)					
Baseline	224.92	36.38	225.02	67.02	0.89*
1 month	229.76	64.33	235.13	42.06	0.87*
3 months	230.10	22.06	226.30	46.50	0.60*
6 months	222.61	46.50	226.03	22.28	0.83*
12 months	235.00	12.11	234.75	23.96	0.87*
*p-*value	0.82^†^		0.88^†^		0.52‡
Subfoveal choroidal thickness (μm)					
Baseline	230.51	95.22	227.94	91.04	0.73*
1 month	246.85	35.40	244.92	76.95	0.88*
3 months	229.10	23.74	230.55	71.00	0.86*
6 months	228.06	79.16	226.84	72.89	0.91*
12 months	224.51	4.30	234.30	54.72	0.66*
*p-*value	0.81^†^		0.84^†^		0.49^‡^

Analysis used *Mann-Whitney U test to compare the groups at each time-point; ^†^Wilcoxon signed-rank test to compare baseline and endpoints within a group; and ^‡^generalized estimating equation to compare changes with time between the groups

*IVD* intravitreal dexamethasone, *RNFL* retinal nerve fiber layer

The bold font for *P*-values indicates a statistical significance (i.e., *P* < .05)

### Changes of visual acuity and visual symptoms over 1 year

The mean BCVA changed from 0.11 ± 0.23 logMAR at baseline to 0.02 ± 0.20 logMAR at 12 months in the observation group (Wilcoxon signed-rank test, *p* = 0.375), and from 0.12 ± 0.33 at baseline to 0.01 ± 0.06 logMAR at 12 months in the IVD-treated group (Wilcoxon signed-rank test, *p* = 0.366), revealing no significant differences between the two groups (generalized estimating equation, *p* = 0.484). All CRVO patients with good VA (higher than 0.20 logMAR) complained of various visual discomforts including blurry vision, cloudy vision, or newly developed multiple blind spots. The symptoms from patients in the observation group improved after 70.7 ± 27.37 days without treatment, whereas IVD-treated patients showed significantly faster improvement in visual symptoms, in 20.7 ± 18.53 days (Mann-Whitney U test, *p* < 0.001).

## Discussion

We retrospectively compared non-ischemic CRVO without ME cases to determine the effect of IVD therapy. This study revealed for the first time that IVD-treated CRVO eyes had greater improvement in venous engorgement, as indicated by improvement in vascular parameters (e.g., tortuosity and width), and reduction of retinal hemorrhage compared to untreated eyes. IVD implantation was also effective in reducing disc swelling, as a more prominent decrease in RNFL thickness was observed in the treated group.

The natural clinical course of the two CRVO types are considerably different: outcome is much better in non-ischemic CRVO than in ischemic CRVO [26]. In our study, retinal venous engorgement, venous tortuosity, and optic disc edema remained after 1 year in 63.6, 54.5, and 36.3%, respectively, of observed CRVO patients without treatment. It is remarkable that venous engorgement/tortuosity was seen only in 30.0% / 22.2% of patients, respectively, and optic disc edema had resolved in all cases of IVD-treated CRVO eyes in our study. According to a large-scale retrospective study, more than 12% of non-ischemic CRVO cases converted to ischemic CRVO [23]. In our study, ischemic conversion was not observed in both treated or untreated CRVO eyes. However, our study did not include a large enough number of patients to demonstrate the effect of IVD on ischemic conversion of CRVO.

Visual prognosis of CRVO has been widely reported to be poor, most commonly caused by ME [26]. However, even without ME, visual loss from CRVO is possible due to macular ischemia, retinal neovascularization, or neovascular glaucoma [12, 27]. CRVO patients in our study also suffered from poor VA at initial visit or complained of severe deterioration of vision quality, even though their visual loss was not severe. There was no significant difference in final VA between the IVD-treated group and the observation group; however, symptomatic improvement was much faster by about 50 days and more complete in the treated group. We expect that the treatment group will be able to show a more significant improvement in VA than in observation group, if we perform a study with a sufficient number of CRVO patients.

For ME secondary to CRVO, previously published studies show that IVD [15, 28] and anti-vascular endothelial growth factor (VEGF) [27, 29–31] treatments are effective in achieving significant improvement in BCVA. However, there have yet not been definitively proven treatments for CRVO eyes without ME. The most recent surgical treatment trials by radial optic neurotomy with pars plana vitrectomy showed controversial results [32, 33]. The theoretical basis for radial optic neurotomy for treatment of CRVO was to relieve pressure at the scleral canal, an optic nerve decompression procedure, thereby relieving ischemia induced by compartment syndrome. Thrombolytic therapies are usually advised to CRVO patients based on evidence of their proven effectiveness in major systemic venous thrombotic disorders. However, Hayreh [12] revealed that improvement in vision and visual field disorder was less likely for aspirin users. Retinal laser photocoagulation is only indicated for neovascularization [34, 35].

The pathophysiology of CRVO is not completely understood. It is believed to result from a blockage of central venous outflow, commonly at the site of optic disc or at arteriovenous crossings, and thrombosis of the main retinal vein is thought to result in CRVO [23, 36]. In elderly patients, venous stasis caused by arteriosclerotic changes of retinal artery, and fibrous tissue envelope is the predominate mechanism for occlusion [37], whereas a hypercoagulable status is the main cause in young patients [38]. However, CRVOs also occurs commonly in healthy patients with no underlying systemic disease. Inflammation and edema of ONH leading to compression of CRV have been suggested as possible causes of CRVO [9, 16]. This may account for the favorable anatomical and functional outcomes in eyes treated with IVD in our study. IVD will act to relieve ONH swelling by providing an anti-inflammatory effect, thereby alleviating ONH edema.

Given the paucity of data regarding the exact pathophysiology of CRVO and its treatment, especially in non-ischemic CRVO without ME, our treatment outcome is encouraging. We speculate that IVD exerts its beneficial effects by the following mechanism. Compartment syndrome at the optic nerve canal could lead to axonal and capillary compression in ONH. The resultant pressure applied to CRV could lead to narrowing of CRV diameter (mechanical compression of CRV) and stasis of venous return from central retinal artery (impairment of venous perfusion) at the lamina cribrosa level. As a result, increased ischemia and release of cytotoxic factors could lead to vasogenic and cytotoxic disc edema, aggravating the compartment syndrome and creating a vicious cycle. This might explain the favorable response to IVD as observed in our study. IVD could counteract both released inflammatory factors and VEGF, thereby reducing disc edema, which would help CRV to restore its original diameter and improve venous return. This explains why patients who received IVD immediately began to show resolution of disc edema along with improvement in retinal hemorrhage and venous tortuosity. These changes, which imply pathophysiologic improvement, are clinically meaningful in terms of lowering the risk of RVO recurrence and secondary complication development.

This study was limited by the small number of included subjects and its retrospective nature. Since we could only analyze indirect changes in impairment of central venous return, direct measurement of venous perfusion was not available. Through prospectively planned studies, an analysis based on fluorescein angiography or OCT angiography should be performed to evaluate the change of retinal perfusion more accurately. As VA only reflects foveal function, assessment of VA alone seems insufficient to compare the visual function between treated and untreated CRVO eyes. The secondary outcome of visual symptoms is subjective, and it may be affected by placebo effects. To determine functional improvement of IVD-treated CRVO, changes in visual field and electroretinography should be analyzed and compared in future prospective studies, as these were not performed in our study.

In summary, IVD implant injections seem to have significant beneficial effects in the improvement of venous engorgement, retinal hemorrhage, and disc swelling in non-ischemic CRVO patients. The action mechanisms of these implants for such pathomorphologic improvements should be studied in more detail, as they may provide additional evidence for treatment use. Further study with more patients is required to reveal whether dexamethasone implants can also provide improvement in visual function, such as retinal sensitivity.

## Conclusion

This retrospective cohort study firstly revealed that IVD implant for the treatment of CRVO without ME was significantly effective in improving venous engorgement, retinal hemorrhage, RNFL swelling, and visual symptoms by presumed alleviation of venous outflow. In two cases treated with IVD implants, a complete resolution of cilioretinal artery occlusion associated with CRVO was observed. Therefore, we concluded that IVD implant may be an effective treatment option in CRVO with no ME and further studies are warranted to verify this.

## Abbreviations

BCVA: Best-corrected visual acuity
CRV: Central retinal veins
CRVO: Central retinal vein occlusion
CVOS: the Central Vein Occlusion Study
DD: Disc diameter
ESR: Erythrocyte sedimentation rate
IOP: Intraocular pressure
IVD: Intravitreal dexamethasone
ME: Macular edema
OCT: Optical coherence tomography
ONH: Optic nerve head
RNFL: Retinal nerve fiber layer
